# Immunosuppression as a Hub for SARS-CoV-2 Mutational Drift

**DOI:** 10.3390/v15040855

**Published:** 2023-03-27

**Authors:** Guy Shapira, Tal Patalon, Sivan Gazit, Noam Shomron

**Affiliations:** 1 Faculty of Medicine, Tel Aviv University, Tel Aviv 69978, Israel; 2 Edmond J. Safra Center for Bioinformatics, Tel Aviv University, Tel Aviv 69978, Israel; 3 Kahn Sagol Maccabi (KSM) Research & Innovation Center, Maccabi Healthcare Services, Tel Aviv 69978, Israel; 4 Maccabitech Institute for Research and Innovation, Maccabi Healthcare Services, Tel Aviv 68125, Israel

**Keywords:** SARS-CoV-2, immunosuppression, mutational drift, COVID-19, infection

## Abstract

The clinical course of coronavirus disease 2019 (COVID-19), caused by severe acute respiratory syndrome coronavirus-2 (SARS-CoV-2), is largely determined by host factors, with a wide range of outcomes. Despite an extensive vaccination campaign and high rates of infection worldwide, the pandemic persists, adapting to overcome antiviral immunity acquired through prior exposure. The source of many such major adaptations is variants of concern (VOCs), novel SARS-CoV-2 variants produced by extraordinary evolutionary leaps whose origins remain mostly unknown. In this study, we tested the influence of factors on the evolutionary course of SARS-CoV-2. Electronic health records of individuals infected with SARS-CoV-2 were paired to viral whole-genome sequences to assess the effects of host clinical parameters and immunity on the intra-host evolution of SARS-CoV-2. We found slight, albeit significant, differences in SARS-CoV-2 intra-host diversity, which depended on host parameters such as vaccination status and smoking. Only one viral genome had significant alterations as a result of host parameters; it was found in an immunocompromised, chronically infected woman in her 70s. We highlight the unusual viral genome obtained from this woman, which had an accelerated mutational rate and an excess of rare mutations, including near-complete truncating of the accessory protein ORF3a. Our findings suggest that the evolutionary capacity of SARS-CoV-2 during acute infection is limited and mostly unaffected by host characteristics. Significant viral evolution is seemingly exclusive to a small subset of COVID-19 cases, which typically prolong infections in immunocompromised patients. In these rare cases, SARS-CoV-2 genomes accumulate many impactful and potentially adaptive mutations; however, the transmissibility of such viruses remains unclear.

## 1. Introduction

Severe acute respiratory syndrome coronavirus-2 (SARS-CoV-2) emerged in late 2019, sparking the coronavirus disease 2019 (COVID-19) pandemic [1] and causing millions of deaths worldwide [2]. The persistence of the virus is partly attributed to its adaptiveness, notably, the emergence of highly divergent viral variants, capable of partial evasion of previously acquired immunity [3]. From the initial origin of the virus [4], to the emergence of highly mutated viral variants [5], the mechanisms underlying the most consequential evolutionary steps taken by SARS-CoV-2 remain uncertain.

The mutational rate of SARS-CoV-2 is deceptively slow. The virus is subjected to strong purifying selection, quickly purging most intra-host single-nucleotide variants (iSNVs) [6], and the iSNVs that remain unpurged are mostly selected against during transmission [7]. An extremely narrow transmission bottleneck limits the time for iSNVs to emerge and the number of virions transmitted, keeping genetic diversity low and restricting the impact of iSNVs on larger evolutionary scales [8,9,10].

In late 2020, following 11 months of unusually weak selection in the initial period of the pandemic [11,12], three highly divergent SARS-CoV-2 variants emerged, marking a major turning point in their evolution and the COVID-19 pandemic. These variants of concern (VOCs) [13] are the product of a drastically different evolutionary process, one that is approximately four times faster [14], strongly adaptive [15], and has the capacity to accumulate multiple epistatic mutations that are individually deleterious [16]. The origin of VOCs remains uncertain, but the leading hypothesis suggests rare, chronic infections in immunocompromised hosts as a likely source [5]. Indeed, some chronic infections have an accelerated mutational rate, greater genetic diversity, and rapid adaptation over a long period of time [17,18]. Adaptive evolution correlates with the duration of infection, the cause of immune deficiency, viral rebound, and exogenous antibody treatment [19,20]. Overall, adaptive evolution is seemingly restricted to rare, extreme cases of profoundly immunocompromised hosts, but it is also affected by commonly variable factors such as age, sex, and infection duration [19].

It is unclear if other host parameters have any effect on the rate or trajectory of SARS-CoV-2 evolution.

In this study, we use SARS-CoV-2 genome sequencing data paired with the electronic health record of its host in order to test the potential association between host clinical parameters and viral intra-host evolution.

## 2. Materials and Methods

### 2.1. Data Sources and Collection

#### 2.1.1. Data Sources

Anonymized electronic health records were retrieved from the centralized computerized database of Maccabi Healthcare Services (MHS). MHS is a state-mandated, not-for-profit health fund in Israel that insures and provides healthcare services to 26.7% of the Israeli population. The MHS computerized database has been maintained centrally for over thirty years. This enabled a comprehensive longitudinal medical follow-up, including demographic data, clinical measurements, outpatient and hospital diagnoses and procedures, medications dispensed, imaging performed, and comprehensive laboratory data from a single central laboratory.

#### 2.1.2. Data Collection

COVID-19-related information consisted of vaccination dates and the results of any PCR tests for SARS-CoV-2. COVID-19-related hospitalizations and mortality records were retrieved as well. COVID-19-related data also included the viral sequencing of some of the PCR tests, which were chosen randomly by MHS. Additionally, data comprised information on chronic conditions from MHS’s automated registries, including immunocompromised conditions, cardiovascular diseases, hypertension, diabetes mellitus, chronic kidney disease, chronic obstructive pulmonary disease, and obesity (defined as a body mass index of 30 kg/m^2^ or higher). The study population included all MHS members who had both a positive PCR test result and viral sequencing information from that test.

#### 2.1.3. Collection, Extraction and Sequencing of RNA Samples

RNA was extracted from nasopharyngeal swabs using a Biomek i7 automatic liquid handler (Beckman Coulter, Brea, CA, USA) by magnetic bead separation (RNAdvance Viral XP Reagent Kit, Beckman Coulter, C59543) according to the manufacturer’s instructions. Briefly, 200 µL of sample was taken directly from the testing tube and added to a 2 mL tube containing 150 µL of lysis buffer (Beckman Coulter) in a BSL2 biological hood. Samples were kept at RT for 20 min to allow viral inactivation and proper lysis, and subsequently loaded to the liquid handler for reformatting into a 96-wells deep-well plate. Three hundred and fifty microliters of magnetic beads (RNAdvance Viral Bind-VBE) was added to each sample. Thereafter, the plate was incubated for 5 min to allow the binding of the magnetic beads to the RNA. After binding, the plate was automatically transferred to an on-deck 96-well magnet (Magnum FLX^®^, Alpaqua, Beverly, MA, USA) for 5 min to allow bead settling. Samples were washed twice with 80% ethanol (Biolabls, Israel) and eluted in molecular-grade water (Biolabls, Israel). Purified RNA was kept in a 96-well format plate at −80°.

SARS-CoV-2 whole-genome libraries were prepared using the Illumina COVIDSeq protocol according to the manufacturer’s protocol (Illumina Inc., San Diego, CA, USA). The first strand synthesis was carried out on RNA samples isolated using the RNAdvance Viral XP Reagent Kit. The synthesized cDNA was amplified using a multiplex polymerase chain reaction (PCR) protocol, producing 98 amplicons across the SARS-CoV-2 genome (https://artic.network; accessed on 15 December 2022). The PCR-amplified product was later processed for tagmentation and adapter ligation using IDT for Illumina Nextera UD Indexes Set A, B, C, and D (384 indexes, 384 samples). Further enrichment and cleanup were performed as per protocols provided by the manufacturer (Illumina Inc.). All samples were processed as batches in a 384-well plate that consisted of one of the COVIDSeq positive control HT (CPC HT), two no template control (NTC), and one negative sample. Finally, these 384 libraries were pooled together into 8 pools of 48 samples each. Pooled samples were quantified by the Qubit 4.0 fluorometer using an HS DS DNA kit (Invitrogen Inc., Carlsbad, CA, USA), and fragment sizes were determined by the TapeStation 4150 via a DNA HS D1000 kit (Agilent). The pooled libraries were further normalized to a 4 nM concentration, and 5 μL of each normalized pool was combined in a new microcentrifuge tube. For sequencing, pooled libraries were denatured and neutralized with 0.2 N NaOH and 400 mM Tris-HCl (pH 8). Dual-indexed paired-end sequencing with 149 bp read length was carried out on the NextSeq 550 platform (Illumina Inc.).

### 2.2. Processing of Whole-Genome Viral Sequencing Data

Raw sequencing data were trimmed using fastp 0.21.0 [21], aligned with bwa-mem2 2.2.1 [22] to the MN908947.3 SARS-CoV-2 reference assembly, and then underwent primer trimming (all the samples were prepared with Artic V3 primers), variant calling, and consensus sequence generation by iVar 1.3.1 [23]. Assignment to phylogenetic clades and quality control were performed by Nextclade 2.10.1 [24], with sequences missing over 3000 nucleotides removed from further analysis. Only positions covered by over 85% of samples with a depth of at least 20 reads are included in the analysis, totaling 25,611 positions. To avoid confounding by the variability of sequencing depth and coverage, variant calling results were downsampled to a uniform depth of 20, in accordance with the method presented in Zhao et al. [25]. Annotations were obtained from the 2019nCoVR database [26]. Phylogenetic reconstruction was performed by the full Nextstrain v11.0 pipeline [27], with the global open-data sequences used for context (https://registry.opendata.aws/ncbi-covid-19, accessed on 20 February 2023).

### 2.3. Genomic and Phylogenetic Annotations

To classify viral mutations as either intra-host (iSNVs, emerging in a host over the course of infection) or inter-host (transmitted from a previous host), we relied on the type of mutation and its relation to phylogenetically proximal viral sequences (50 genome sequences with the shortest phylogenetic distance). In short, iSNVs were defined as mutations with a low allele frequency (present in less than 90% of the reads covering its locus) that were not called in ancestral viral sequences, or rare mutations (<0.05% occurrence in 2019nCoVR) absent from other viral sequences in the clade. The time to most recent common ancestor (TMRCA) was estimated using Nextstrain v11.0 [27], with the default maximum likelihood model and 95% confidence intervals.

#### Statistical Modeling

A generalized Poisson model was used for modeling intra-host variability and testing against individual clinical parameters. The number of iSNVs in each viral genome was set as the dependent variable. The independent variables were selected and tested systematically from the available electronic health records data. To ensure that our results were not skewed by host-independent factors, we considered the date of collection and viral clade as possible confounders. Sequencing coverage remained negatively correlated with the number of overall iSNVs and was thus added to the model as an additional confounder.

### 2.4. Limitations

Researching SARS-CoV-2 intra-host evolution was methodologically challenging and limited the scope of analysis.

The primary limitation of our study is the lack of multiple (longitudinal) viral samples from individual participants over the course of infection. Our method for classifying iSNVs relies on statistics from large-scale surveillance databases and the tendency of iSNVs to produce a small set of mutations that are highly enriched in iSNVs. Furthermore, our samples were generally taken shortly after infection, whereas samples taken during late infection are expected to contain more iSNVs [7], which may be more influenced by host–pathogen interactions. Comparisons with low statistical power were excluded. Single mutation association tests were avoided due to the potential for false positives caused by the founder effect [28].

### 2.5. Ethics Declaration

This study was approved by the MHS Institutional Review Board. Due to its retrospective design, informed consent was waived, and all identifying details of the participants were removed before computational analysis.

## 3. Results

### 3.1. Data Overview

The cohort is composed of 1304 Israeli COVID-19 patients with a positive SARS-CoV-2 polymerase chain reaction (PCR) test result between 27 March 2020 and 22 July 2021 (Table 1). One sample was collected from each participant and sequenced, yielding the whole genome of the infecting virus.

SARS-CoV-2 genomes generally belonged to the commonly circulating viral clades in Israel at the time of sampling. The earliest viral samples, collected towards the end of 2020, were split between the Alpha clade and various pre-VOCs, with the alpha clade becoming dominant in the beginning of 2021. As 2021 went on, pre-VOCs became increasingly uncommon, and more VOCs were observed (Beta, Epsilon, and Mu), but none became dominant. Around June of 2021, the Delta variant emerged and gained near-complete dominance within a short time span.

### 3.2. The mutational Landscape of SARS-CoV-2

**Figure 1:**
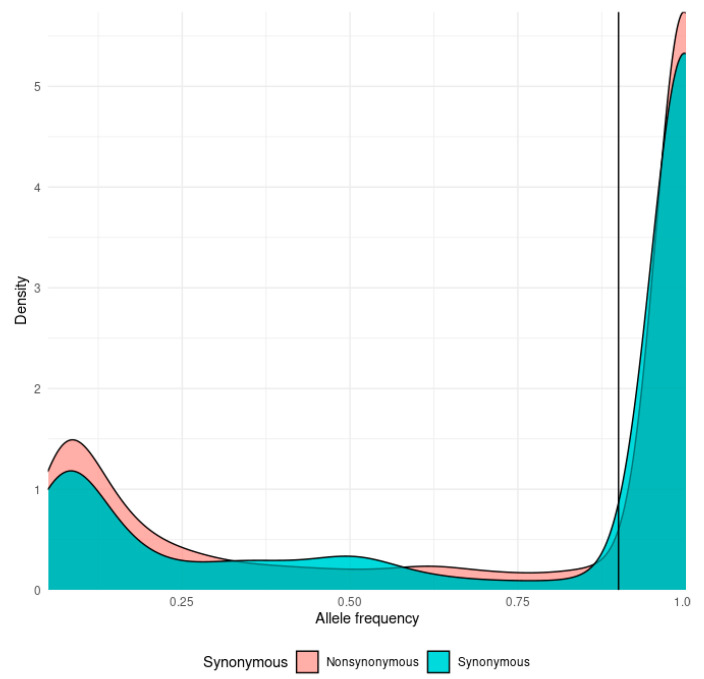
Density plot of the distribution of allele frequencies for synonymous (cyan) and nonsynonymous (orange) mutations. The mutation class threshold (90%) is marked by a vertical line.

**Figure 2:**
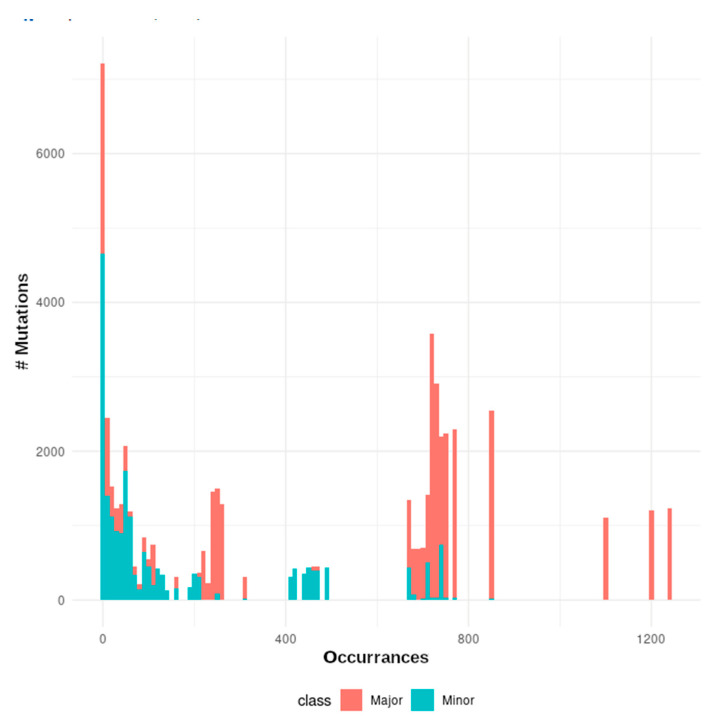
A histogram of the number of mutations (y-axis) by the number of occurrences in the cohort (x-axis), colored according to their assigned class.

**Figure 3:**
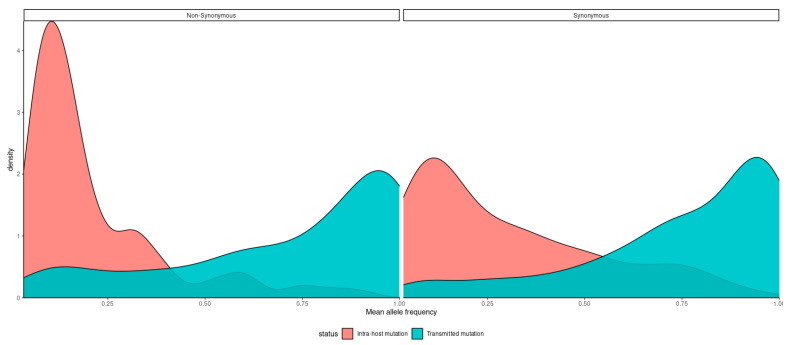
A density plot of transmitted (orange) and intra-host (cyan) mutations by their mean allele frequency, split into synonymous (**right**) and nonsynonymous (**left**) mutations.

**Figure 4:**
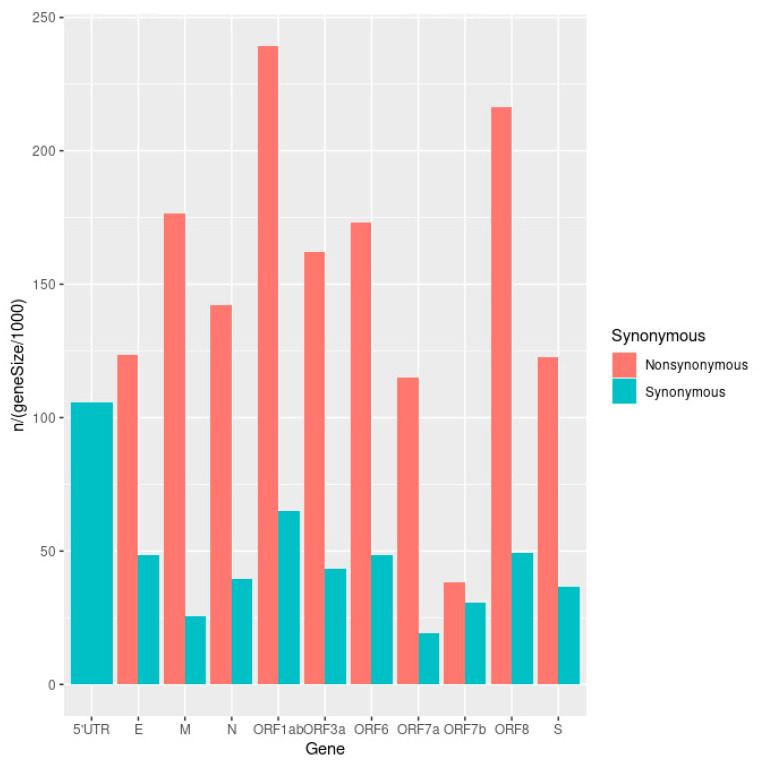
A bar plot of the number of iSNVs in each gene, corrected for gene length and colored according to type (non/synonymous).

Mutations found in SARS-CoV-2 sequencing data were generally split into major “fixed” mutations, found in over 90% of all reads mapped to a locus, and minor mutations, which were found in less than 25% of reads. Ninety percent (90%) allelic fraction was selected as a reasonable cutoff between mutational classes (Figure 1). We find a strong dichotomy in the pattern of transmission, with minor mutations having fewer occurrences and being much less likely to be transmitted (Figure 2 and Figure 3). Mutations were almost always consistent in their class affiliation, with no observations of a mutation switching classes by becoming fixed or unfixed. Consistent with previous observations, nonsynonymous iSNVs were predominantly minor and greatly outnumbered synonymous iSNVs across the viral genome (Figure 3 and Figure 4) [7].

### 3.3. Modelling SARS-CoV-2 Intrahost Diversity

**Figure 5:**
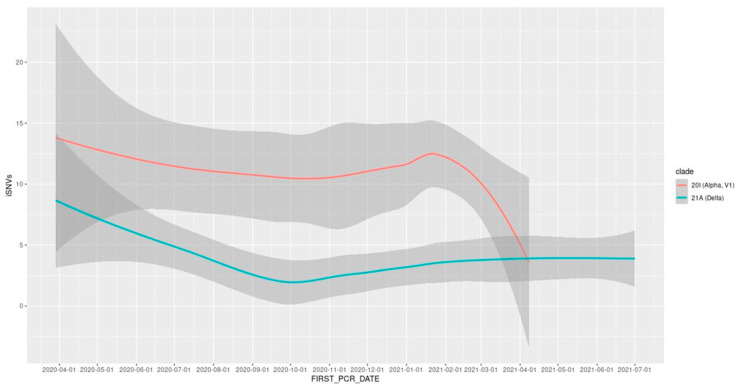
LOESS trend line depicting the change in intra-host diversity over time, by viral clade.

The abundance of iSNVs differed between viral clades; the delta variant showed lower intra-host diversity than did the alpha variant (mean ± SE 3.6 ± 0.4 vs. 11.1 ± 0.9 iSNVs per sample). For both viral clades, iSNVs decreased in diversity as the viral population reached stability (Figure 5). These global changes in the SARS-CoV-2 mutational profile led to an overall decrease in intra-host diversity, which was associated with time (*p* < 2 × 10^−8^; Z = −5.6) and with the gradual replacement of the alpha variant with the delta variant (*p* < 7 × 10^−13^; Z = −7.1).

Taking into account time of sample collection and phylogenetic clade, iSNVs are significantly more abundant in vaccinated hosts (*p* < 1 × 10^−7^; Z = 5.3) and older persons (Z = 4; *p* < 6 × 10^−5^), and significantly less abundant in current smokers (Z = −4; *p* < 4 × 10^−6^). Immunosuppressed hosts and persons with diabetes showed a slight, albeit insignificant, decrease in overall iSNVs (Z = −1; *p* = 0.1 and *p* = 0.16, respectively). Associations were not found between iSNVs and a patient’s sex or disease severity (*p* > 0.2).

### 3.4. The Identification of an Unusual Viral Genome from an Immunosuppressed Host

**Figure 6:**
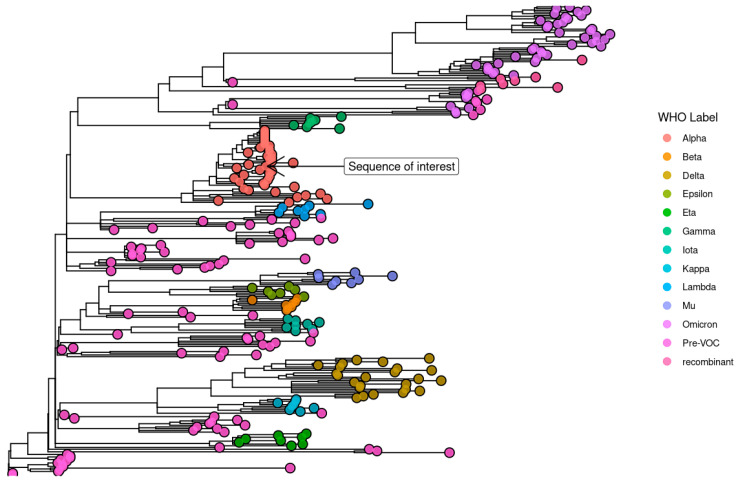
Reconstructed, time-resolved phylogeny of the SARS-CoV-2 genome sequence of interest, obtained from an immunocompromised patient (marked). Tree leaves are colored according to phylogenetic assignment, with X-axis representing time.

One viral genome had an excess of fixed, rare mutations, harboring many iSNVs. We highlight the SARS-CoV-2 genome of the Alpha variant (B.1.1.7) obtained from an immunosuppressed woman in her 70s who first tested positive for infection on 24 February 2021, 37 days after receiving a second dose of the BNT162b2 vaccine (Figure 6). She has been immunosuppressed since 2014 and has minimal change disease and Hashimoto’s thyroiditis. She is treated regularly with L-Thyroxine and steroids. COVID-19 symptoms were mild, and the infection was resolved within a month from initial detection without the need for extra care. About a month late, she reported post-infection fatigue.

The sequence was marked problematic by quality control routines for having 40 mutations which were not shared by any of its ancestors. Phylogenetic analysis estimated the divergence of the virus at 15 December 2020 (95% maximum likelihood CI: 13 November 2020–19 January 2021), meaning that all newly emerged mutations likely accumulated in the span of about two months.

**Figure 7:**
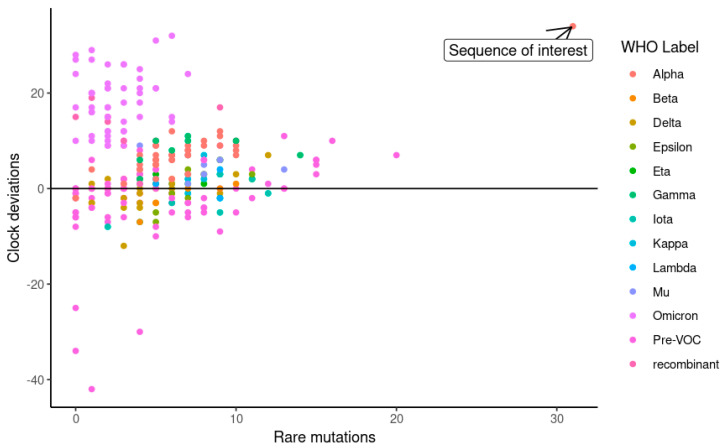
A scatter plot of SARS-CoV-2 genomes included in the reconstructed phylogeny, with the sequence of interest marked. Points representing viral genomes are colored according to phylogenetic assignment, with X-axis representing the number of rare mutations (mutations exclusive to the genome sequence that do not define any major lineage) and Y-axis as an indication of a sequence deviation from the expectation of the phylogenetic model (see Section 2).

The excessive number of mutations in the sequence of interest deviates from the expectations of the model used for phylogeny, having accumulated a large number of mutations in a short time span (Figure 7).

The emerging mutations are considered rare both in the reference phylogeny and in the global phylogeny, with the fixed mutations: NSP3:P1787S, NSP6:G48V, and ORF3a:G11*, found in less than 0.01% of all sequenced genomes.

We identified a stop gain mutation in the beginning of the ORF3a gene, leaving only the first 10 amino acids of a total 275 in the ORF3a protein (G25423T; ORF3a:p.11G>*) (Table S1).

The mutation is completely fixed (present in all 3117 reads covering the locus) and was not identified elsewhere in the dataset; the mutation was reported in only two other samples (according to GISAID; 10 April 2022). Due to the fixation of the mutation and the location of the premature stop codon upstream of all functional domains, a complete loss of function in ORF3a is expected.

## 4. Discussion

### 4.1. A Weak Association between SARS-CoV-2 Intra-Host Diversity and Host Factors

Our analysis of SARS-CoV-2 intra-host diversity suggests only slight differences in the abundance of iSNVs, relative to a host’s age, vaccination status, and smoking habits. The relatively weak association between host factors and the intra-host mutation rate is consistent with previous findings. However, our analysis indicates a positive correlation with age, while the longitudinal study by Li and colleagues found a slight negative correlation [7].

We estimate that the intra-host differences might be indicative of differences in host-pathogen interactions, yet the transmissibility and impact of these differences on overall viral evolution are questionable, owing to strong purifying selection [6]. The nonsynonymous iSNVs were almost exclusive to the lower end of allelic frequency (Figure 3), and no evidence was found of fixation of any iSNVs in the cohort in the wider viral population.

### 4.2. A Case of Chronic SARS-CoV-2 Infection in an Immunocompromised Woman with Hashimoto’s Thyroiditis

A single immunosuppressed woman, who had Hashimoto’s thyroiditis and minimal change disease, harbored a highly unusual viral genome. We estimated the time to most recent common ancestor (TMRCA) to be 2 months prior to sample collection and about a month before vaccination.

There were no other detectable cases of chronic infection in the cohort, no other sequence strongly deviated from the molecular clock estimate and had an excess of rare mutations (Figure 7). This case has multiple characteristics predictive of adaptive evolution: the host is an immunocompromised older woman, treated with steroids and infected for over a month [19]. There was no evidence of transmission from the chronically infected host and no indication whether the hyper-mutated virus remained transmissible.

The cohort included 29 other cases of infection in immunocompromised hosts, some with even more profound immune dysfunction, but none had a remarkable viral genome or an infection longer than a month. While it is possible that chronic infection is a rarity, even among immunocompromised patients, we cannot entirely rule out the possibility of an infection becoming chronic. It is not uncommon for a chronic infection to rebound after a period of low-to-undetectable viral load and for the host to remain asymptomatic and untested [19].

It is possible that long infection and adaptive evolution are more common in hosts with a viral co-infection [29] or as a response to antiviral therapy [30]; however, no such cases were found in this cohort.

### 4.3. Rare, High Impact Mutations of Nonstructural Proteins and Replication Organelle Constituents in Chronic Infection

The viral genome sequenced from the chronically infected host has an excess of chronic mutations affecting non-structural proteins: NSP3, NSP4 and NSP6, all three are essential components of the membrane-bound, SARS-CoV-2 replication organelle [31]. NSP3 bears the rare substitution P1787S, overlapping the Y domain (Y3), involved in the structure of viral RNA-containing double-membrane vesicles (DMV) and suggested to interact with host proteins associated with protein degradation [32]. NSP4 has a string of rare substitutions including L438K, T439H and a stop gain in amino acid 440. NSP4 forms a complex with NSP3 [31] and induces a pro-inflammatory response in the host cell [33]. NSP6 is affected by the extremely rare substitution G48V (present in less than 0.01% of sequenced viral genomes). The substitution affects the C-terminal domain of NSP6, responsible of host DFCP1 recruitment and localization in the host cell [31].

While the importance of these newly emerging mutations in nonstructural proteins cannot be determined with confidence, the density of mutations and their fixation in the intra-host population suggest that several loci were subjected to strong positive selection. The affected amino acids are highly conserved, and their function is essential to viral replication [31], implying that their rarity is due to deleteriousness outside of few unusual cases.

### 4.4. ORF3a Truncation in Chronic Infection

Truncating mutations were previously reported in SARS-CoV-2 accessory proteins, such as ORF7a and ORF8 [34,35]. However, the truncated portion of ORF3a that we identified is much larger and located directly upstream to one of the most conserved loci in the viral genome, where the occurrence of a loss of function mutation is not as common [36]. As the premature stop codon is upstream from all functional domains, with the remaining peptide cleaved from the protein product, ORF3a^Δ^ is predicted to cause complete loss of function of the protein. Two out-of-frame ORFs were identified downstream of ORF3a^Δ^; however, these cannot compensate for the truncation of the canonical ORF, as the peptides produced are non-functional [37]. Due to the rarity and predicted deleteriousness of the mutation, combined with the overall unusual characteristics of this viral genome, we hypothesize that the mutation occurred within the host and that its emergence can be attributed to the immunosuppressed state.

ORF3a is the largest SARS-CoV-2 accessory protein and has a diverse array of functions in the host cell, including subcellular localization to the plasma membrane and Golgi, induction of apoptosis and inhibition of interferon signaling [38,39,40,41]. ORF3a induces incomplete autophagy and then subverts the formation of the autolysosome [42,43]. In addition to the functions of the canonical ORF3a protein, three overlapping protein-coding genes (named ORF3b, ORF3c and ORF3d) were more recently identified and found to contribute to immune evasion; however, the extent of their functions remains mostly unknown [44].

Nemudryi et al. identified a truncating mutation in ORF7a that eliminated translation of a viral transmembrane domain, and that limited replication and viral immune suppression [34]. Like ORF3a^Δ^, the ORF7a truncation is hypothesized to have originated from an immunosuppressed host and to have limited spread. Unlike ORF3a^Δ^, the ORF7a truncation circulated in a small local community for about 1.5 months before disappearing. Based on this comparison and the importance of ORF3a to the viral process, we hypothesize that ORF3a^Δ^ is exceptionally deleterious, possibly to the point of being endemic to severely immunodeficient hosts.

Unlike the slow accumulation of immunity-evasive spike mutations that have been observed in immunosuppressed hosts [45], ORF3a^Δ^ is a rapidly fixated, maladaptive mutation that is likely to impair viral fitness. This largely unknown class of mutations is rarely observed and the extent of its influence on SARS-CoV-2 evolution is unclear.

## 5. Conclusions

In this study, we examined the relation between SARS-CoV-2 evolution and health factors of the human host. Our results suggests that in common acute SARS-CoV-2 infections, viral diversity is only marginally affected by host factors such as age and vaccination.

A second class of SARS-CoV-2 evolution is the chronic infection, found only in a rare subset of immunocompromised hosts and clearly distinct from acute infections in the type of mutations and the rate in which they accumulate. The single case of chronic infection found in our cohort was completely distinct from the rest, with an excess of rare mutations implying a radical difference in selective pressure compared to the common cases. While the association between intra-host evolution and basic clinical parameters requires further examination, our results indicate that the evolutionary leaps that characterize VOCs might be confined to rare and exceptional cases of chronically infected immunocompromised hosts.

## Figures and Tables

**Table 1:** Characteristics of the cohort.

Group	Immunocompetent (N = 1165)	Immunosuppressed ᵃ (N = 30)
Males (fraction)	692/1165	20/31
Age, mean ± SE	35.6 ± 0.5	60.8 ± 3.1
Diabetes mellitus ᵃ, %	7.20%	41.90%
Vaccinated ᵃ, %	90.40%	96.70%
Smoker ᵃ, % (N.A. %)	10.4% (24%)	6.4% (0%)
Alpha variant (fraction)	693/1165	22/30
Delta variant (fraction)	236/1165	4/30
Other VOC (fraction)	7/1165	0/30
Pre-VOC (fraction)	199/1165	4/30

ᵃ Clinical and lifestyle parameters at the diagnosis of SARS-CoV-2 infection. SE: standard error; N.A.: not available.

## Data Availability

According to the Israel Ministry of Health regulations, individual-level data cannot be shared openly. Specific requests for remote access to de-identified community-level data or code used should be referred to KSM, Maccabi Healthcare Services Research and Innovation Center.

## Supplementary Materials

The following supporting information can be downloaded at: https://www.mdpi.com/article/10.3390/v15040855/s1, Table S1: Complete table of mutations identified in the sequence of interest.
